# Emergency care facility access in rural areas within the golden hour?: Western Cape case study

**DOI:** 10.1186/1476-072X-14-5

**Published:** 2015-01-16

**Authors:** Marianne Vanderschuren, Duncan McKune

**Affiliations:** Centre for Transport studies, Department of Civil Engineering, University of Cape Town, Private Bag X3, Rondebosch, 7700 South Africa

**Keywords:** Road Safety Risk, Road crashes, Golden Hour, South Africa

## Abstract

**Background:**

Road Safety is a major cause of death around the world and South Africa has one of the highest road fatality rates. Many measures, engineering and medical, are investigated. However, analysis of the accessibility of emergency care facilities is often overlooked. This paper aims to fill the gap between pre-crash engineering solutions and literature on trauma injuries and emergency care procedures. The focus is on the role that accessibility to emergency care facilities in rural areas plays, given that 50% of the world’s population lives in rural areas, which are often omitted from international research. The Western Cape (a rural province with low population volumes and high volume roads in South Africa) is analysed as an example of access to trauma care in rural areas.

**Method:**

It is internationally accepted that the time to emergency care facilities influences the survival chances. However, the international literature still debates the exact time period. In this paper, the ‘Golden Hour’ is used to analyse the accessibility of emergency care facilities in rural areas and establish a geographical analysis method which identifies risk areas. The analysis can be repeated if the international literature debates regarding the exact time period changes.

**Results:**

A Geographical Information System (GIS) tool revealed that 53% of the fatalities in the rural parts of the Western Cape occur outside the Golden Hour. In high risk crash areas, the fatality risk is up to nine times higher than the province’s rural average.

**Conclusions:**

People in need of trauma care after a road crash are most likely to survive if they receive definitive care timeously. At the time of the study, the rural areas in the Western Cape had 44 Emergency Medical Services stations and 29 medical facilities that can assist to provide definitive (trauma) care. Further optimisation of the facility locations is recommended and research has begun.

More advanced geographical modelling is possible when improved data becomes available on the ‘Golden Hour’ theory, differential times for varying injury types or travel speeds of ambulances. This, more advanced, modelling can reduce the road crash burden in rural areas around the world further.

## Background

Launched on 11 May 2011, the Decade of Action has the official goal of ‘stabilising and then reducing’ global road traffic fatalities by 2020. The global plan for the Decade of Action is organised around 5 pillars of the ‘Safe Systems’ approach. The pillars include: road safety management, infrastructure (addressed in pre-crash engineering solutions), safe vehicles, road user behaviour and post-crash care (international literature on trauma injuries and emergency care procedures). This advisory plan, endorsed by governments (including national, provincial and local governments in South Africa), UN agencies, multilateral institutions and NGOs coming together in the UN Road Safety Collaboration, is providing inspiration and guidance for many countries and organisations working to reduce road safety casualties (http://www.fiafoundation.org/our-work/road-safety-fund/un-decade-of-action).

In March 2013 the World Health Organization (WHO) published the *Global Status Report on Road Safety 2013: Supporting a Decade of Action* [1]. The report presents 2010 data for 182 participating countries and provides a baseline for monitoring the Decade of Action for Road Safety (2011–2020). About 1.24 million road traffic deaths occurred throughout the world in 2010, indicating a plateau since the publication of the first status report in 2009. The African region has some of the world’s highest road traffic fatalities making it the 9th leading cause of death in the region [2]. Death and injury on the roads in these countries exacerbates poverty by depriving households of the main income earner, and imposing a heavy burden on health services. Some 50% of all fatalities are amongst vulnerable groups (e.g. pedestrians, cyclists and public transport users). By 2030 it is predicted that road crashes will be the 5th leading cause of death, unless serious attention is given to addressing the root causes.

The risk of dying as a result of a road traffic collision is highest in the African Region at 24.1/100 000 population (the global rate is 18/100 000) according to Peden [3]. Nigeria and South Africa have the highest road traffic fatality rates (33.7 and 31.9/100 000, respectively) and, together with the Democratic Republic of Congo, Ethiopia, Kenya, Tanzania and Uganda, account for 64% of all road traffic deaths in the region. Overall, the African region has less than 2% of the world’s registered vehicles, but almost 20% of the global traffic deaths. An 80% increase in traffic deaths between 2000 and 2020 has been predicted by Kopits and Cropper [4].

Traditionally, engineers analyse pre-crash conditions categorised into six E’s, which include Engineering, Education, Enforcement, Evaluation, Environment and Encouragement. More often than not, post-crash conditions are not taken into account by engineers when analysing fatalities and looking for measures to implement in order to reduce the casualty rate, as they are assumed to be part of the medical research field. Although this is true, the period between a crash and emergency care is overlooked in many regions, and can play a major role in rural areas, such as rural US, EU, South America, Asia and Sub-Saharan Africa. The analysis in this study focuses on the post-crash phase of road-traffic crashes, attempting to fill the identified knowledge gap.

There is a phenomenon in trauma care known as the ‘Golden Hour’ , which is commonly used to characterise the urgent need for the care of trauma patients. This term implies that morbidity and mortality are affected if care is not instituted within the first hour which occurs immediately after injury [5]. Aid during this period has been shown to reduce mortality rates dramatically according to Muckart [6]. Trunkey [7] states that: ‘head-injured patients must receive surgery within four hours of injury, while those with severe haemorrhage require surgical intervention within 20 minutes”. Lerner and Moscati [5] point out the lack of definitive scientific evidence regarding the ‘Golden Hour’ theory and request further studies, but indicate that the general principal that time plays a role is widely accepted. This study, therefore, accepts the concept and uses 60 minutes to demonstrate the geographical analysis concept. A large part of the Golden Hour, in which it is essential to commence effective treatment of seriously injured patients in order for them to survive, is usually taken up by the travel time to and from a crash scene [8], indicating the need for this study.

In the 2010/11 period, the Road Traffic Management Corporation (RTMC) reported that there were 1 026 fatal crashes, resulting in 1 258 fatalities in the Western Cape [9]. This is 9% of the 13 802 fatalities that occurred throughout South Africa in the 2010/11 period. About 80% of all crashes in the Western Cape happen within the City of Cape Town, which is home to about 80% of the province’s population. However, fatalities in the province provide a different view. Around 50% of all fatalities occur in the rural parts of the province [10]. Besides the higher average speeds, which increase the crash impact, the hypotheses is that rural victims often do not receive appropriate care within the Golden Hour.

Road safety is dynamic in its approach and new ideas, such as identifying the accessibility of emergency care facilities, need constant exploring and analysis. For the analysis in this paper, the fatalities data that was included was obtained from the iPAS fatalities database. A total of 7210 fatalities that occurred in the 2000–2007 period were mapped and analysed to calculate the likelihood that rural road crash victims are able to receive appropriate medical trauma care within the Golden Hour.

It must be noted that throughout the paper, a road crash fatality is defined as a death occurring within a six day period of the crash. The accuracy of the data regarding the six day period is unknown to the authors.

## Methods

Given the high level of fatalities in South Africa and the Western Cape, and anecdotal information that rural hospitals can, sometimes, not handle the number of injured road crash victims needing trauma care, the Western Cape was identified as a suitable case for this study.

An extensive literature review regarding the various concepts of pre- and post-trauma care was conducted. Various articles were researched from journals such as Accident Analysis and Prevention, Annals of Emergency Medicine, International Journal of Health Geographics, etc. On completion of the literature review, a research gap was identified: very limited research had been conducted into the accessibility of emergency care facilities. Having identified the research gap, a model was developed to simulate the travel times, travel patterns etc. of ambulances, in order to assist with the analysis of post-crash trauma care. ArcGIS was chosen to develop the model, as it is an appropriate and available tool at the University of Cape Town.

The data required for the model was identified and gathered from various sources in both the engineering and medical fields. Table 1 shows the data that was utilised, in addition to the source of the data. Once collected, the data was validated by choosing samples and checking the validity of these samples. In some instances, the required data was not available (e.g. the average speeds that ambulances travel at). In these instances assumptions were made that were reasonable according to various experts (e.g. the ambulances travelled at the speed limit).

**Table 1:** **Data Utilised in the Study**

Data	Description	Source
Crash locations	Shapefile containing locations of crash locations, in addition to other related information.	iPAS database (PGWC)
EMS stations	Shapefile containing locations of EMS stations, in addition to other related information	PGWC
Medical facilities	Shapefile containing locations of medical facilities, in addition to other related information	PGWC
Road network	Shapefile containing road network information for the Western Cape (no speed limits)	Previous research conducted
Road network speed limits	Shapefile containing road network information for the Western Cape (with speed limits)	PGWC

Numerous analyses were run, based on adaptations in the modelling approach, due to increasing accuracy in the assumptions, particularly those relating to the travel patterns of ambulances. Tools within ArcGIS Network Analyst were used to run the analyses. Initially, the Service Area tool was used to simulate the travel times of the ambulance. On completion of the analysis, it was found that the analysis procedure did not reflect reality. This is because both the EMS stations and the ‘definitive care’ medical facilities were used as both start and end locations. This is incorrect as in reality, EMS stations will be used as start locations and ‘definitive care’ medical facilities will be used as end locations. However, the Service Area tool is capable of calculating these conditions. Therefore, the Closest Facility tool was utilised.

The Closest Facility function finds the quickest route from a facility to an incident, and then records the travel time. The Closest Facility function, therefore, needed to be run twice: once from the nearest EMS station to the accident location, and second from the accident location to the nearest ‘definitive care’ medical facility. The results of both outputs were combined in Excel, along with the dispatch times and loading times. The total mission time was then obtained, and a process was run in order to check which fatalities were inside or outside of the ‘Golden Hour’. The final Excel output was then used as an input into ArcGIS.

Once the analysis phase was completed, critical zones were identified. In addition, the effect of various factors on the service areas was investigated. On identification of the risks, possible mitigation measures were recommended based on current pre-crash road safety systems and post road-crash trauma care systems.

## Results

### Identifying access to trauma care

**Figure 1:**
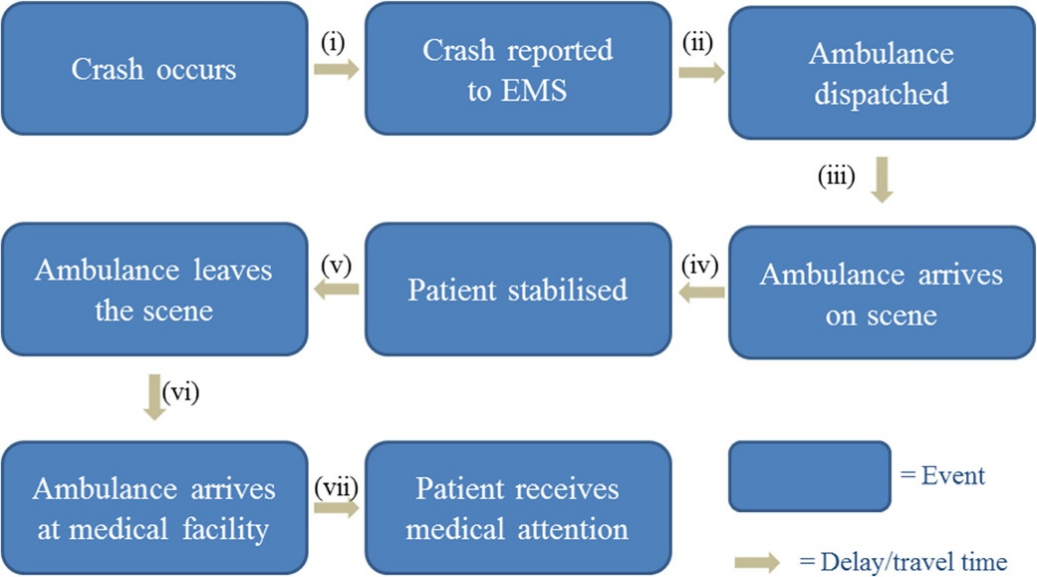
**Sequence of post-crash events.**

As indicated, engineers mostly analyse pre-crash conditions categorised into six E’s and the accessibility to emergency care facilities, especially in rural areas, is often overlooked. Figure 1 gives the events in chronological order. Information needed for assessing the serviceability of a medical facility, is the time it takes between each of the post-crash events shown (i.e. the arrows). The summation of the time it takes between each post-crash event must not exceed the ‘Golden Hour’. The events were adapted from a study performed by Gitelman into Safety Performance Indicators for trauma management in Europe [11], based on the identification of local emergency procedures.

The arrows in Figure 1 represent the time taken between each of the post-crash events. The seven arrows represent the following times:

i. **Notification time:** the time it takes from when a crash occurs until it is reported to Emergency Medical Services (EMS). This time can vary drastically depending on the consciousness of those involved in a crash, the presence of any witnesses, and the availability of a landline or mobile service;
ii. **Dispatch time:** the time taken between the EMS call centre receiving the emergency call and the ambulance being dispatched;
iii. **Travel time (a):** the time it takes for the ambulance to travel to the scene of the crash;
iv. **Stabilisation time:** the time taken by the paramedics to stabilise the patient;
v. **Loading time:** the time taken to load both the patient and any equipment used;
vi. **Travel time (b):** the time it takes for the ambulance to travel to a medical facility; and
vii. **Admission time:** the time taken between arrival at the hospital and the patient receiving the required medical attention.

In order to do an analysis for the whole of the Western Cape, the summation of the times between each post-crash event needs to be calculated for each medical facility. Of the seven time sequences discussed, three were obtained directly from the Provincial Government of the Western Cape (PGWC). These three time sequences include the dispatch time, the stabilisation time and the loading time. Table 2 shows the dispatch time and on-scene time (defined as the loading time plus the stabilisation time) per region, as well as an overall average. These times were obtained from the 2012 transport related incidents to which ambulances responded.

**Table 2:** **Average time sequences per region**

Region	Dispatch time (minutes)	On-scene time (minutes)
Cape Winelands	0.10	24.46
Central Karoo	0.05	32.25
Eden	0.16	20.25
Overberg	0.22	28.26
West Coast	0.79	33.62
Overall	0.26	27.55

Assuming the best case scenario, i.e. that the notification time and the admission time are zero, the remaining time can be split into travel times a and b. Overall, there are 32.19 minutes of the Golden Hour unaccounted for, which leaves an average of 16.09 minutes for each component of travel time. The analysis also included variations of travel times, with a maximum of 32.19 minutes in total for both directions.

### Medical care facilities

**Figure 2:**
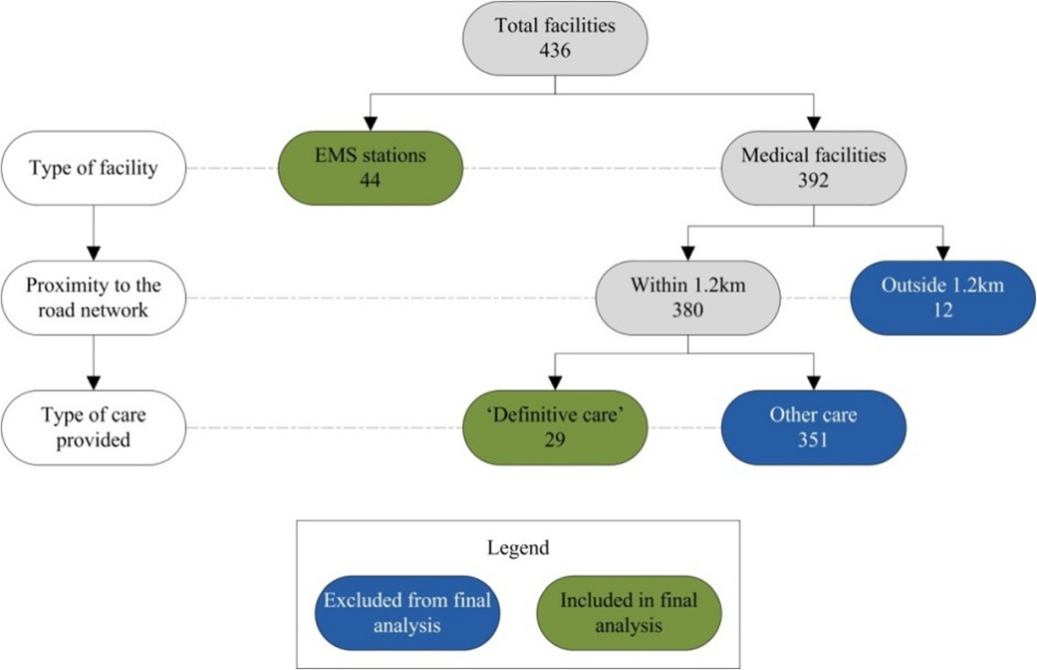
**Breakdown of included medical facilities.**

The medical facilities that were used in the analysis were obtained from the PGWC. The total number of medical facilities within the study area is 392 (excluding EMS stations). However, not all of these facilities provide ‘definitive care’ , which in discussions with an official from the PGWC, was defined as ‘the completion of required treatment’. Figure 2 provides an overview of the validation process for ‘definitive care’ facilities. In the final analysis, 44 EMS stations and 29 definitive care facilities, operational at the time of this study, were included.

### Golden Hour service gaps

All information required for the identification of the area that falls outside the Golden Hour service (i.e. Golden Hour Service Gaps) was now available.

The following data was included in the analysis:

- ‘Definitive care’ medical facilities (29 in total);
- All EMS stations (44 in total);
- The overall average dispatch time of 0.26 minutes was used for all five regions;
- The overall average on-scene time of 27.55 minutes was used for all five regions; and
- Hence, a maximum combined travel time to and from the crash scene of 32.19 minutes.

Of the 20 981.5 km of road analysed, 14 682.4 km (70.0%) of road falls outside of the Golden Hour.

### Definitive care crashes

**Figure 3:**
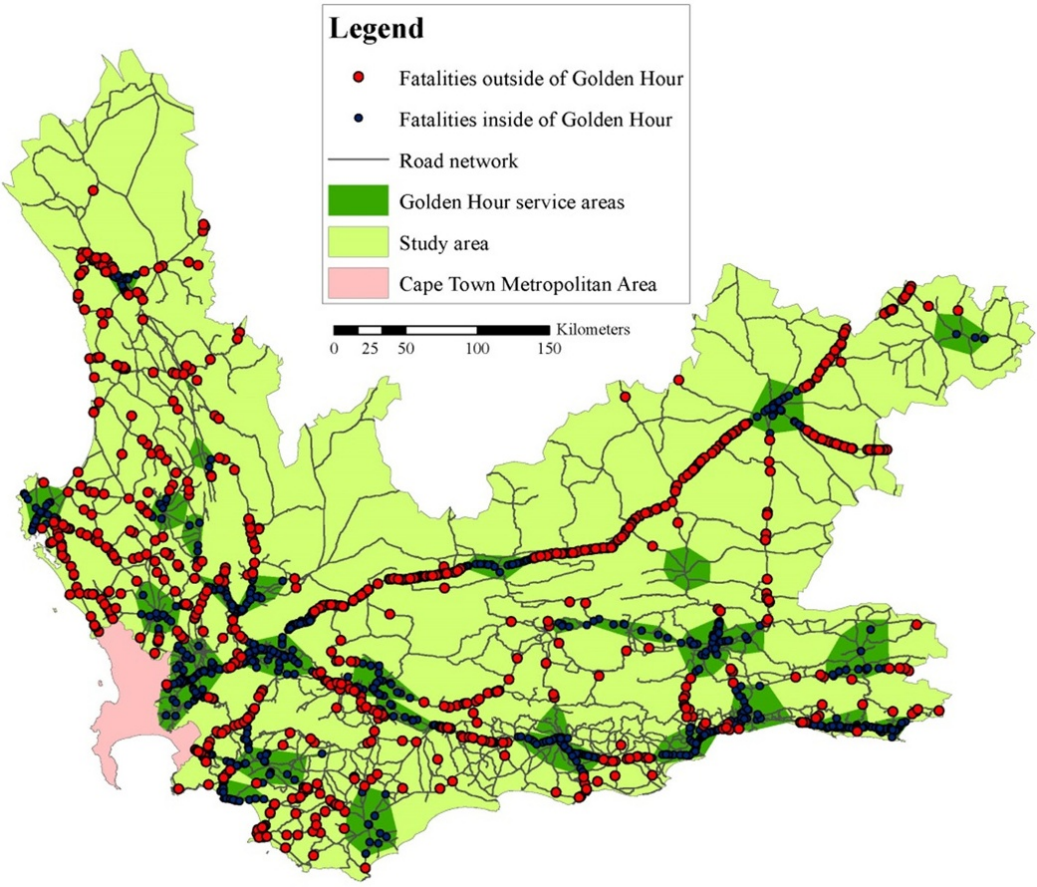
**Access to definitive care in the Western Cape.**

For the actual fatalities in the Western Cape, there are multiple theoretical possibilities to access definitive care. Various analyses were carried out using the ‘Closest Facility’ function on ArcGIS, as discussed in the Methods section. It was found that, of the 7 210 fatalities that occurred in the 2000–2007 period, 3 825 (53.1%) of the fatalities were outside of the Golden Hour (see Figure 3).

### Hazardous location identification

Hazardous zones in the Western Cape were identified in a study called ‘Safely Home – Baseline’ [10]. The study revealed nine hazardous zones. A total of 4 022 fatalities or 55.8% of all rural fatalities in the Western Cape (including some rural towns), were situated within these hazardous zones.

**Figure 4:**
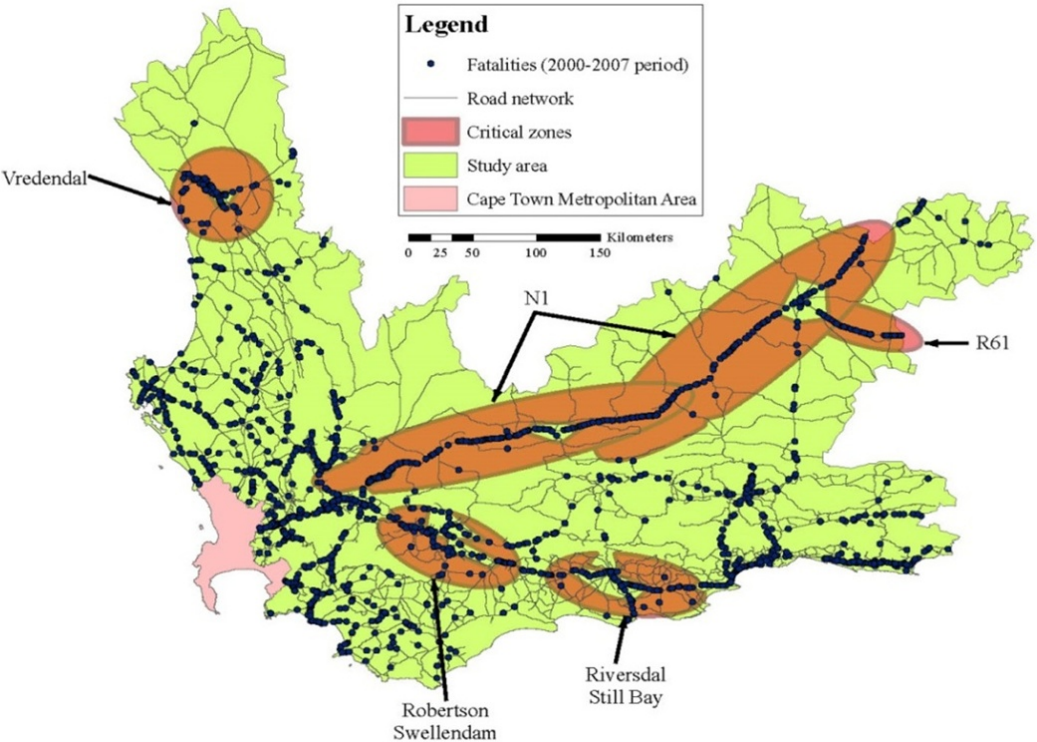
**Critical Zones Identification.**

The next step was to overlay hazardous zones and service gaps, to enable the identification of critical zones (see Figure 4).

Four of the nine hazardous zones were not identified as critical zones, as the majority of the fatalities in the four zones were within the ‘Golden Hour’. For the other five critical zones (see Figure 4), a total of 1 934, or 26.8%, of the fatalities from the 2000–2007 period were found within these areas. The total length of road that is found in critical zones is 671.3 km, which is 3.2% of the road network.

### Fatality rates

When looking at the fatality rate, the average for the whole study area is 0.34 fatalities/km. However, when analysing the hazardous zones, the fatality rate is 2.48 fatalities/km, which is 7.3 times higher than the average. Within critical zones, the fatality rate is higher, i.e. 2.88 fatalities/km, which is 8.5 times higher than the average.

One of the major roads, National Route 1 (N1), used in the province to reach commercial destinations (i.e. the Gauteng province, including Pretoria and Johannesburg) and holiday destinations (people visiting Cape Town) was analysed separately. The N1 has a length of 562.5 km and is host to 1 776 fatalities - a fatality rate of 3.16 fatalities/km. This is higher than the average critical zone, and is 9.3 times higher than the average. It was expected that the service gaps on the N1 would have a higher fatality rate than the service areas on the N1. However, it was found that they both had the same fatality rate (3.16 fatalities/km).

The authors were surprised by the last finding and attempted to identify the reason for the lack of difference between the service areas and service gaps. The population densities along the N1 route between Beaufort West and Paarl vary significantly from 1 700 people/km^2^ in Paarl, to 7.8 people/km^2^ in Laingsburg. A higher population density suggests that there are more causes for crashes. These causes include increased pedestrian activity in combination with speed differentials. Therefore, a higher number of crashes should imply a higher number of fatalities. The pedestrian fatalities are concentrated in the densely populated areas (in particular De Doorns and Paarl). When excluding the pedestrian fatalities from the total fatalities, there is a significant change in the fatality rates. The fatality rate within the service areas is 2.25 fatalities/km, and within the service gaps the fatality rate is 2.91 fatalities/km. This finding supports the notion that the pedestrian crashes have an impact on the fatality rates in densely populated areas and that, for non-pedestrian fatality rates, they are significantly different between service areas and service gaps.

### Positioning of emergency medical services stations

The effectiveness of an EMS station is dependent on its position relative to a medical facility that provides ‘definitive care’. The analysis revealed that 10 EMS stations do not fall within service areas. This is because there are no ‘definitive care’ medical facilities within the Golden Hour reach of an EMS station.

## Conclusions

It was established that people in need of trauma care, after a road crash, are most likely to survive if they receive definitive care within the first hour after the crash occurred. In this study, high risk areas, falling outside the ‘Golden Hour’ service area were identified for the rural parts of the Western Cape. This study demonstrates the benefits of a geo-analysis of this kind. Similar analyses are recommended for rural areas around the world.

South Africa has an extremely high fatality rate. Although measures are taken to reduce these high fatality rates, this rate seems to stay amongst the highest in the region (31.9/100 000 inhabitants). In the rural parts of the Western Cape, nine hazardous zones were identified (host to 55.8% of all fatalities in the rural parts of the Western Cape) that need further, detailed investigation.

The process which is followed, within the Western Cape, to get trauma patients to a definitive care facility was established. It was found that only 32.19 minutes are available for travel (in total for both directions). Through the use of a GIS tool, including the EMS and definitive care facilities, an analysis was carried out, identifying geographical areas where trauma patients can receive the required care within the so called Golden Hour (service areas). A total of 53.1% of all crashes happen in the service gaps (outside the Golden Hour).

When overlapping the hazardous zones with the service gaps, four critical zones were identified. The National Road 1 (N1) is the largest and most critical area. In this study, the chances of survival were, therefore, ranked according to fatality rates (fatalities/km), including the service areas and N1 corridor. Table 3 summarises the fatality rates for the areas that were investigated. It is clear that the fatality rates in the hazardous zones and service gaps are a multitude of the average for the study area. For the N1, population densities and the presence of pedestrians appear to influence the results.

**Table 3:** **Fatality rates on the N1 (Western Cape)**

Location	Fatality rate (fatalities/km)	Ratio to the average
Study area (average)	0.34	1.0
Hazardous zones	2.48	7.3
Critical zones	2.88	8.5
N1 service areas	3.16	9.3
N1 service gaps	3.16	9.3
N1 service areas (excluding pedestrians)	2.25	6.6
N1 service gaps (excluding pedestrians)	2.91	8.6

Given the results of this analysis and the limited number of EMS services and medical facilities, it is recommended to investigate if the optimisation of resources is possible. This, however, falls outside the scope of this study.

**Figure 5:**
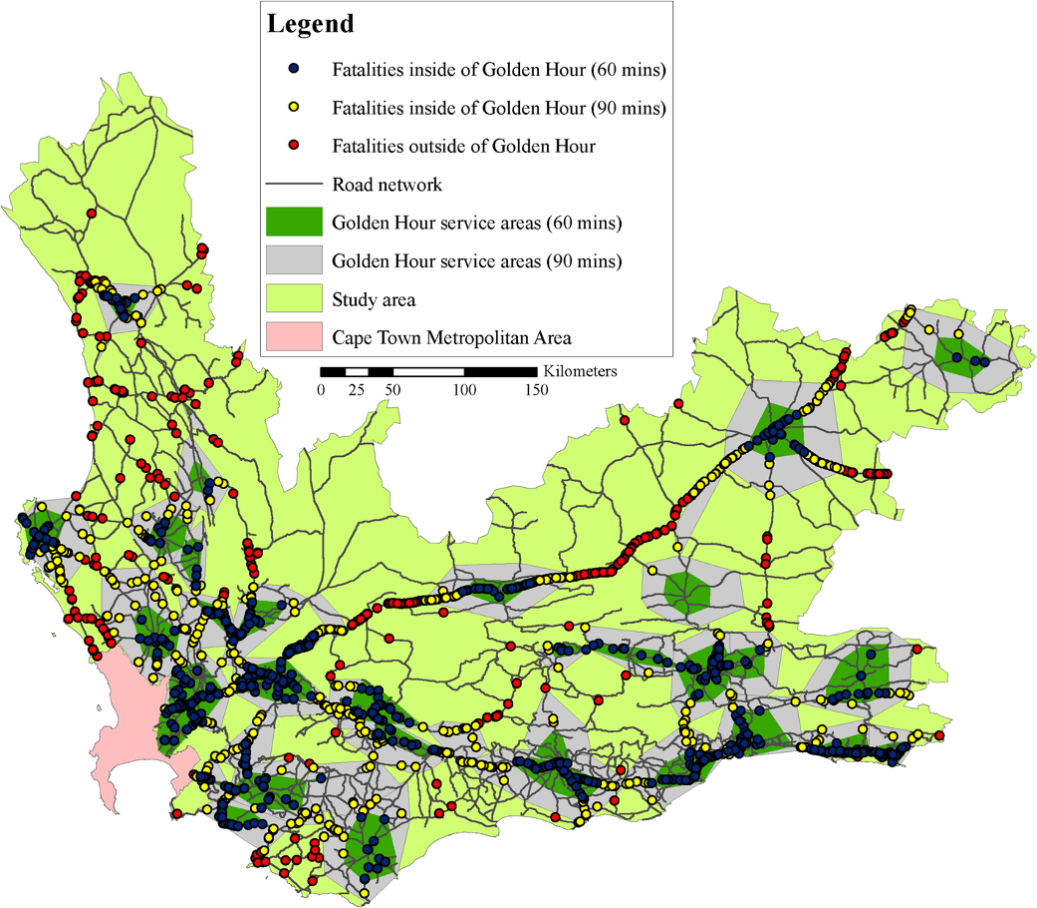
**Extended ‘Golden Hour’ Coverage.**

The international literature still debates the ‘Golden Hour’ theory. Furthermore, some literature indicates that the time to trauma care differs, depending on the injury type. Figure 5 shows the inclusion of 90 minute ‘Golden Hour’ service areas, resulting in a 30.2% reduction in the number of fatalities in the service gaps (i.e. from 53.1% to 22.9%), showcasing the fact that the study can be adopted to include differing times etc. It is recommended to repeat and fine tune this study, when new concepts are accepted in the medical literature.

It should also be noted that travel times determined by the model can be improved with increasing accuracies in the data inputs. Improvements in the tracking of ambulances in the study area will lead to better knowledge on the average speeds travelled by ambulances, and when these improvements are made, it is recommended that the study be repeated.
